# The Impact of miRNAs in Health and Disease of Retinal Pigment Epithelium

**DOI:** 10.3389/fcell.2020.589985

**Published:** 2021-01-15

**Authors:** Daniela Intartaglia, Giuliana Giamundo, Ivan Conte

**Affiliations:** ^1^Telethon Institute of Genetics and Medicine, Naples, Italy; ^2^Department of Biology, Polytechnic and Basic Sciences School, University of Naples Federico II, Naples, Italy

**Keywords:** miRNAs, retinal pigment epithelium, RPE differentiation, miR-204, miR-211, AMD

## Abstract

MicroRNAs (miRNAs), a class of non-coding RNAs, are essential key players in the control of biological processes in both physiological and pathological conditions. miRNAs play important roles in fine tuning the expression of many genes, which often have roles in common molecular networks. miRNA dysregulation thus renders cells vulnerable to aberrant fluctuations in genes, resulting in degenerative diseases. The retinal pigment epithelium (RPE) is a monolayer of polarized pigmented epithelial cells that resides between the light-sensitive photoreceptors (PR) and the choriocapillaris. The demanding physiological functions of RPE cells require precise gene regulation for the maintenance of retinal homeostasis under stress conditions and the preservation of vision. Thus far, our understanding of how miRNAs function in the homeostasis and maintenance of the RPE has been poorly addressed, and advancing our knowledge is central to harnessing their potential as therapeutic agents to counteract visual impairment. This review focuses on the emerging roles of miRNAs in the function and health of the RPE and on the future exploration of miRNA-based therapeutic approaches to counteract blinding diseases.

## Introduction

In vertebrates, the RPE originates from the dorsal portion of the optic cup, while the retina and the optic stalk develop from the distal/ventral portion (Amram et al., 2017). Once specified, the RPE cells begin to differentiate, changing in size and shape. With the folding of the optic cup, RPE progenitor cells constitute a ciliated and pseudostratified epithelium, which is committed into a cuboidal epithelium following the formation of interphotoreceptor matrix. As eye morphogenesis proceeds, the presumptive RPE tissue assumes an apical to basolateral polarity and forms both microvilli and tight junctions. On further differentiation, the RPE cells adopts a hexagonal shape, with elongated microvilli and a smooth basal surface which makes continuous contact with its basement membrane (Marmorstein et al., 1998). In adults, RPE tissue comprises a monolayer of polarized and pigmented epithelial cells that resides at the interface between the light-sensitive outer segments of the PR and vessels of choriocapillaris (Strauss, 1995, 2005). The RPE layer is a critical constituent tissue capable of absorbing the light energy focused by the lens on the retina, thus mitigating photo-oxidative stress (Bok, 1993). In addition, the RPE represents part of the tight retinal–blood barrier (Simo et al., 2010); supports the isomerization of all-*trans*-retinal to 11-*cis*-retinal, the visual chromophore required for photoreceptor excitability (Strauss, 2005); protects from oxidative stress (Strauss, 2005); secretes growth factors that help to maintain the structural integrity of photoreceptors and choriocapillaris endothelium (Strauss, 2005; Mazzoni et al., 2014); establishes ocular immune privilege by expressing immunosuppressive factors (Ao et al., 2018); and finally is critical in the continuous renewal of the photoreceptors outer segments (POS), which are regularly shed by phagocytosis, to rebuild light-sensitive outer segments from the base of the photoreceptors (Mazzoni et al., 2014). To realize these complex functions, intricate molecular networks, activated by extracellular and intracellular signals, are simultaneously employed to maintain RPE homeostasis and function. Due to the significant activity of microRNAs (miRNAs) in modulating essential biological processes by targeting networks of functionally correlated genes, it is unsurprising that miRNAs have emerged as indispensable components of these molecular networks in the RPE (Soundara Pandi et al., 2013; Greene et al., 2014). Importantly, alterations to gene regulatory networks controlling any of the above activities of the RPE can lead to degeneration of the retina and loss of visual function. This in turn gives rise to diseases including retinitis pigmentosa, age-related macular degeneration (AMD), and other blindness conditions in humans (Strauss, 2005; Amram et al., 2017).

### miRNAs Biogenesis and Function

Identified in the early 1990s in *Caenorhabditis elegans* (Lee et al., 1993; Wightman et al., 1993), miRNAs are a class of small (typically 20–26 nucleotides) non-coding RNA molecules that regulate gene expression at the posttranscriptional level, in a well-characterized process in which the miRNAs bind to target sites in the messenger RNA. miRNA biogenesis [reviewed by Ha and Kim (2014) and Treiber et al. (2019)] is a complex process that begins with a long primary transcript comprising a stem-loop hairpin structure (pri-miRNA), which is transcribed by RNA polymerase II, capped, and polyadenylated (Treiber et al., 2019). Pri-miRNA subsequently undergoes stepwise processing to produce single hairpins (typically 60–70 nucleotides) referred to as precursor miRNAs (pre-miRNAs) by the RNase III enzyme Drosha and its cofactor DiGeorge syndrome critical region gene 8 (Dgcr8), which compose the “Drosha microprocessor” complex (Hata and Kashima, 2016). Following Drosha processing, pre-miRNA is then exported to the cytoplasm by exportin 5 (Xpo5), where maturation is completed through a second round of stepwise processing by the RNase III enzyme Dicer and its cofactor transactivation-responsive RNA-binding protein (TRBP or PACT). This gives rise to a small RNA duplex (Ha and Kim, 2014). The miRNA duplex is then handed over to a member of the Argonaute (AGO) protein family, which selects one of the strands of this duplex (the guide strand) and discards the other strand (the passenger strand) (Treiber et al., 2019). Finally, the miRNA/AGO complex and the GW182/TNRC6 family of proteins form the active miRNA-induced silencing complex (miRISC), which is recruited to target mRNAs by pairing the “seed” region of miRNA, with a partially complementary sequence in the 3′-untranslated region (3′-UTR) of target mRNAs (Ha and Kim, 2014). Thus, miRNA-guided gene silencing promotes translational inhibition and mRNA decay (Treiber et al., 2019). Most miRNAs are expressed in a highly tissue-specific manner (Wienholds et al., 2005). Importantly, miRNAs have been shown to display either protective or pathological-promoting effects in several tissues. Although the number of miRNAs known to be involved in eye development has increased significantly, a more complete understanding of how miRNAs regulate cellular processes and how their own expression is regulated is yet to be achieved. In the RPE, miRNAs were found to be key regulators of tissue differentiation, homeostasis, and function. In this review, we discuss some of the known functions of the most relevant miRNAs involved in the RPE development, maintenance, and function. A more comprehensive dissection of the roles of miRNAs in the RPE will not only improve our understanding of the molecular networks controlling RPE homeostasis and our diagnostic abilities but will also lay the foundations for studying how miRNAs could act as therapeutic tools for the treatment of ocular diseases.

## miRNAs as Emerging Regulators of RPE Development and Maintenance

The gene regulatory networks involved in establishing the RPE development and differentiation have been extensively studied in a variety of model organisms reviewed in Amram et al. (2017), but the critical roles of miRNAs in these processes have only recently been explored. Recent studies have reported miRNA expression profiles during RPE development and differentiation in a variety of species, using multiple approaches including next generation sequencing (NGS) technology and *in situ* hybridization (ISH) analysis (Deo et al., 2006; Xu et al., 2007; Damiani et al., 2008; Decembrini et al., 2009; Arora et al., 2010; Georgi and Reh, 2010; Hackler et al., 2010; Karali et al., 2010; La Torre et al., 2013; Wohl and Reh, 2016). Importantly, NGS analyses showed dynamic miRNA expression profiles during RPE differentiation, indicating a strict correlation between miRNA expression levels and the stages of iPSC-RPE differentiation (Wang et al., 2014). To characterize the roles of miRNAs in the RPE development, depletion of the enzymes essential for their maturation and processing were examined in several animal models. Conditional knockout (cKO) mice, for Dicer1, Drosha, or Dgcr8 genes in the RPE, demonstrated that this class of proteins is relevant for RPE differentiation and maintenance. Dicer1 cKO in the ocular pigmented cell lineage around postnatal (PN) day 9.5, generated by crossing Dgcr8^LoxP/LoxP^ animals with *Dct-Cre*, *Tyrp2-Cre*, and α*-Cre* mice, provided the earliest evidence of the impact of simultaneous loss of miRNAs in the generation and survival of non-retinal cell types (Davis et al., 2011). Consistent with these findings, Dicer1 cKO at the optic vesicle stage (E9.5), using *Dct-Cre* mice, revealed alterations to RPE differentiation. At PN11 days, *Dicer1^loxP/loxP^/Dct-Cre* mice showed RPE cells that were smaller than normal, depigmented, and failed to express enzymes required for the visual cycle (Ohana et al., 2015; Amram et al., 2017). However, RPE cell fate and hexagonal cell morphology were preserved, as was the conserved expression of transcription factors (i.e., OTX2, MITF, and SOX9), which participate in RPE specification and maintenance (Amram et al., 2017). Similar phenotypes were also observed in both RPE-specific Drosha and Dgcr8 cKO mouse models (Ohana et al., 2015; Amram et al., 2017). Importantly, the maintenance of RPE cell fate was further corroborated by the transcription profile of Dicer1-deficient RPE cells. These data gave rise to the hypothesis that RPE fate might be acquired and maintained through miRNA-independent mechanisms. Together, these findings supported the notion that miRNAs were necessary for the execution of proper differentiation programs during RPE development (Ohana et al., 2015; Amram et al., 2017). In contrast to the findings on the role of Dicer1 in miRNA biogenesis during RPE differentiation, a non-canonical Dicer1 activity was demonstrated to participate in the adult RPE from *Dicer1*^loxP/loxP^/*Best1-Cre* mice, with *Cre* recombinase expression beginning at P10 and peaking at P28 (Iacovelli et al., 2011). In differentiated RPE cells, a primary role for Dicer1 was documented in the degradation of toxic transcripts of Alu transposable elements, rather than miRNA biogenesis (Iacovelli et al., 2011). Supporting this notion, single miRNA-binding protein knockout mice, for Ago1, Ago3, Ago4, or Tarbp2, did not reveal RPE morphological alterations (Sundermeier and Palczewski, 2016). Consistent with these findings, AAV-mediated delivery of a Best1-Cre recombinase expression cassette into mice carrying conditional Drosha, Dgcr8, or Ago2 alleles did not show any defects in RPE morphology (Sundermeier and Palczewski, 2016). However, lack of miRNA biogenesis in both differentiating and adult RPE commonly affected the homeostasis and survival of the adjacent photoreceptor cells. Nevertheless, to gain deeper phenotypic understanding, additional studies should analyze alternative miRNA biogenesis mechanisms and the compensatory molecular networks that counteract lack of enzymes essential for miRNA maturation and processing in the RPE (Yang and Lai, 2011).

### The Impact of miRNAs on RPE Differentiation, Proliferation, and Migration

**FIGURE 1:**
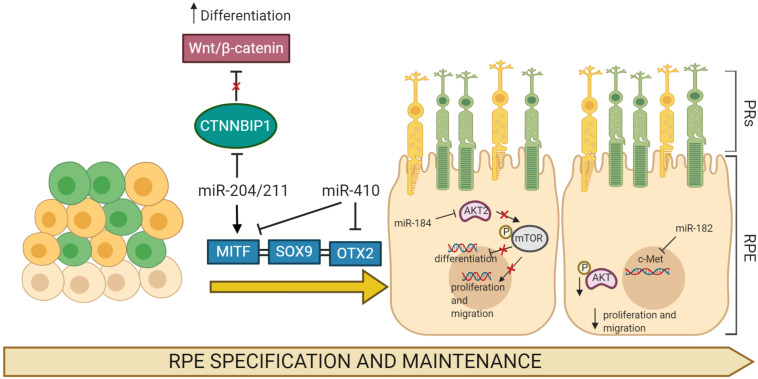
MicroRNA (miRNA)-regulated gene networks involved in RPE specification and maintenance. Simplified schematic of a gene network regulating development, differentiation, and migration in RPE cells. Key genes in these processes, *MITF*, *SOX9*, and *OTX2*, are all regulated by specific miRNAs that modulate their expression profiles. The expression of multiple miRNAs increases and/or affects the processes of RPE differentiation, proliferation, and migration through the regulation of target gene mRNAs.

Notably, in a few cases, the roles of individual miRNAs have been dissected in fish, frogs, and mice, demonstrating the relevance of these small non-coding RNAs in RPE development. The members of miR-204/211 family are arguably the most extensively studied miRNAs in the RPE. These miRNAs are identifiable from their seed regions and are highly enriched in the developing and differentiated RPE. Overexpression of miR-204/211 in human fetal RPE (hfRPE) cells has been shown to effectively counteract a lack of MITF, a key regulator of RPE differentiation, and has been reported to rescue RPE de-differentiation phenotype (Adijanto et al., 2012). Similarly, studies in human parthenogenetic embryonic stem cells (hPESCs) and hfRPE demonstrated that miR-204 upregulation contributes to RPE differentiation program by repressing the target gene *CTNNBIP1*, an inhibitor of the Wnt/β-catenin pathway (Li et al., 2012). Interestingly, although both miR-204 and miR-211 were required for RPE differentiation, deletion of only one member of this miRNA family resulted in no visible alterations to RPE differentiation, suggesting possible redundancy of function, at least during RPE development *in vivo*. The role of miRNAs in RPE differentiation was also confirmed through studies on miR-184 in hiPSC-RPE and zebrafish, demonstrating the role of miR-184 in controlling RPE differentiation. Downregulation of dre-miR-184 suppressed the expression levels of RPE markers, while its overexpression stimulates RPE development by inhibiting the AKT2/mTOR pathway (Jiang et al., 2016). Further supportive evidence for the role of miRNAs in RPE differentiation programs has been reported in additional studies. miR-20b/106a and miR-222/221 families have been described to regulate RPE differentiation by modulating the expression levels of several transcription factors including Sox9, Otx1, Pax6, and Meis2 (Ohana et al., 2015). Among the miRNAs downregulated during RPE differentiation, both miR-302d-3p and miR-410 are prominently repressed during RPE development. Importantly, overexpression of miR-302d-3p induced hiPSC-RPE de-differentiation and impairment of cellular phagocytosis and promoted cell-cycle progression, via repression of both *TGF-*β*R2* and p21^Waf^^1/Cip1^, a cyclin-dependent kinase inhibitor, encoded by *CDKN1A* (Li et al., 2012; Jiang et al., 2018). Importantly, the inhibition of miR-410 facilitated RPE differentiation in both AESCs and umbilical cord blood-derived mesenchymal stem cells (UCB-MSCs) by derepression multiple RPE development-relevant genes, such as *RPE65* and *OTX2* (Choi et al., 2015, 2017). Moreover, multiple studies have reported that inhibition of RPE proliferation and migration were induced by increased expression miR-451a (Shao et al., 2019) and miR-218 (Yao et al., 2019). On the other hand, an overproliferative effect of miR-182 (Figure 1) was reported to downregulate the p-Akt pathway in RPE via repression of *c-Met* expression (Wang et al., 2016a). Similarly, overexpression of miR-34a in cultured subconfluent ARPE-19 cells displayed remarkable inhibition of *c-Met*, in turn reducing *HGF/SF* expression and preventing cell proliferation and migration (Hou et al., 2013). Moreover, besides these well characterized miRNAs, additional RPE-enriched miRNAs have recently emerged as regulators of RPE development (Table 1). However, their capacities to alter RPE cell differentiation, proliferation, and migration and the identification of their targets and regulatory networks remain to be solved.

**TABLE 1:** Selected miRNAs involved in the regulation of RPE development, differentiation, and migration.

miRNA	Target	Function	References
miR-204	PAX6, MITF, CTNNBIP1	Positive regulation of RPE development and differentiation	Conte et al. (2010); Adijanto et al. (2012), Li et al. (2012)
miR-184	AKT2/mTOR	Induction of RPE differentiation	Jiang et al. (2016)
miR-410	RPE65, OTX2, LRAT, MITF	Inhibition of RPE development and differentiation	Choi et al. (2015, 2017)
miR-196a	PAX6, SIX3, LHX2, CHX10	Negative regulation of RPE development	Qiu et al. (2009)
miR-182	MITF, c-Met	Negative regulation of RPE proliferation and migration	Wang et al. (2016a)
miR-302d	CDKN1A, TGFβR2	Induction of RPE de-differentiation	Li et al. (2012); Jiang et al. (2018)
miR-183/-96/-182	OTX2, NRL, PDC, DCT	Triggering of neuronal cells differentiation	Davari et al. (2017)
miR-27b	NOX2	Negative regulation of RPE proliferation and migration	Li et al. (2018)
miR-34	c-Met, LGR4	Modulation of RPE proliferation and migration	Hou et al. (2013, 2016)
miR-451a	ATF2	Inhibition of RPE proliferation and migration	Shao et al. (2019)
miR-218	RUNX2	Inhibition of RPE cells proliferation	Yao et al. (2019)
miR-26b	Unknown	RPE cell proliferation promotion	Zhang et al. (2017b)
miR-203a	SOCS3	Regulation of RPE differentiation	Chen et al. (2020b)
miR-194	ZEB1	Inhibition of EMT of RPE cells	Cui et al. (2019)
miR-148	Unknown	RPE EMT promotion	Takayama et al. (2016)
miR-124	RhoG	Control of EMT	Jun and Joo (2016)
miR-125b/let-7a	Unknown	Promotion RPE fate during differentiation	Shahriari et al. (2020)
miR-29b	AKT2	Regulation of RPE EMT	Li M. et al. (2016)
miR-328	PAX6	Promotion of RPE proliferation	Chen et al. (2012)
miR-21	TGFβ	Cell proliferation and migration promotion	Usui-Ouchi et al. (2016)

## RPE Homeostasis and Function

The RPE exhibits common features of an epithelium tissue, preserving the structural and physiological integrities of adjacent tissues. The RPE plays crucial roles in retinal homeostasis, including the formation of the outer blood–retinal barrier (BRB), through junctional complexes, which prevents the entry of toxic molecules and plasma components into the retina (Rizzolo, 2007). Alterations to RPE physiology and homeostasis result in photoreceptor death and blindness. As part of the BRB, the RPE transports water, ions, and metabolic waste from the subretinal space to the choriocapillaris (Marmor, 1990; Hamann, 2002), where it takes up nutrients such as glucose or vitamin A, retinol, and fatty acids to the photoreceptors (Ban and Rizzolo, 2000; Marmorstein, 2001). The RPE secretes different growth factors according to variable physiological and pathological conditions. During eye development, growth factors released from RPE play key roles in choroidal neovascularization. To regulate transport across the RPE, numerous pumps, channels, and carriers are specifically distributed to either the apical or the basolateral membrane. Transporters and channels with roles in the selective transport to and from the choroid vasculature are located in the basal RPE. The apical side of the RPE projects long thin and sheet-like microvilli into the interphotoreceptor matrix. The long microvilli engulf the tips of the rod and cone photoreceptor outer segments; this ensures that the segments are orientated in the appropriate plane for optics and maximizes the cellular surface for efficient epithelial transport. The short microvilli mainly function in the turnover of POS through phagocytosis. Moreover, RPE cells are constantly exposed to oxidative stress due to exposure to light and the generation of reactive oxygen species (ROS) following phagocytosis of POS (Plafker et al., 2012). A robust endogenous antioxidant defense mechanism is therefore critical. This is characterized by the production of endogenous antioxidants such as glutathione, whose production decreases with age and/or incidence of degenerative diseases (Beatty et al., 2000). Oxidative stress alters RPE integrity and promotes the activation of the inflammation system, activating complementary cascades and upregulating the production of cytokines and chemokines. Notably, the mature RPE itself maintains tissue homeostasis through long-term cell survival, with little evidence of cell turnover and a stem cell compartment for *de novo* cell production. This static homeostasis suggests that as RPE cells age, substantial tissue changes occur that compromise RPE function and morphology, triggering causes of AMD (Fuhrmann et al., 2014). Based on its structural characteristics, the RPE plays crucial roles in retinal homeostasis, of which a primary role is the formation of the outer BRB that prevents the entrance of toxic molecules and plasma components into the retina (Rizzolo, 2007). Breakdown of the BRB can lead to visual loss in a number of ocular disorders, including diabetic retinopathy (DR).

### MicroRNAs Are the “Traffic Wanders” of the RPE Homeostatic Center

**FIGURE 2:**
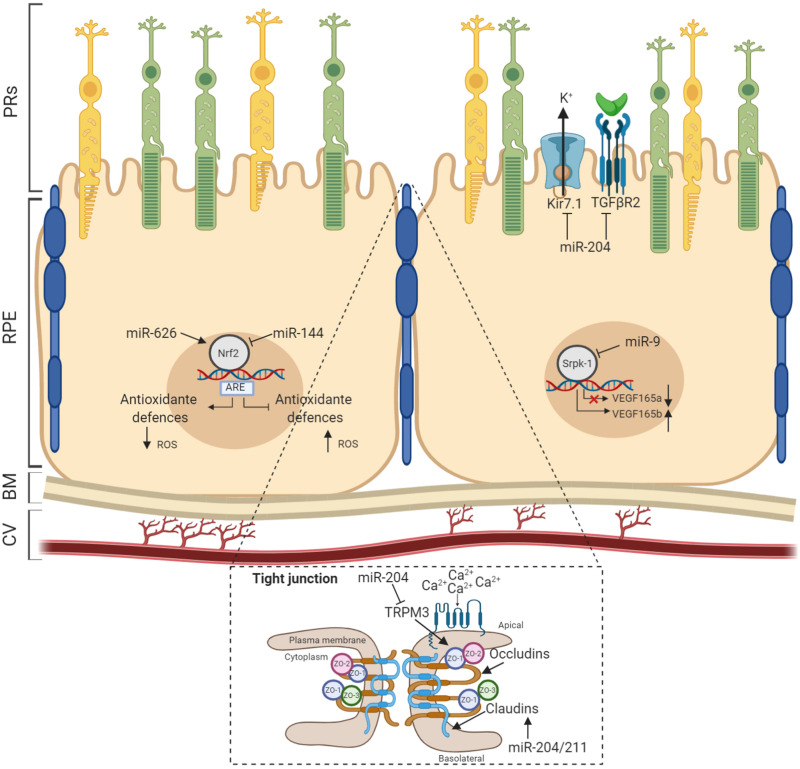
MicroRNAs (miRNAs) act as modulators of RPE homeostasis. Several miRNAs have been demonstrated to influence the correct functioning of the RPE homeostatic machinery. Recognized miRNAs species associated to specific RPE functionality are shown with validated targets. BM, Bruch’s membrane; CV, choroidal vasculature.

Maintenance of RPE layer integrity is controlled by junctional complexes, characterized by the presence of multiple proteins including occludin, claudins, and junctional adhesion molecules. This type of cell–cell adhesion establishes a barrier between the subretinal space and choriocapillaris (Harhaj and Antonetti, 2004). The first evidence for the role of miRNAs in RPE homeostasis was reported from studies on both Dicer and Dgcr8 cKO mice, where ultrastructural analysis of RPE revealed disorganized basal infoldings with large cytoplasmic vacuoles and debris accumulation at the interface of Bruch’s membrane, resulting in atrophy and disruptions to the integrity of the RPE (Sundermeier et al., 2017; Wright et al., 2020). Furthermore, miRNA studies on miR-204 revealed its prominent functional role in the formation and maintenance of the epithelial barrier of the RPE. Wang et al. (2010) demonstrated that miR-204 significantly alters claudin 10 and 19, tight-junction proteins both highly expressed in human RPE, while miR-204/211 affects transthyretin (TTR) secretion, an important marker for epithelial barrier integrity and critical for vitamin A transport across the RPE (Cichon et al., 2014). In agreement with this, overexpression of the miR-204/211 family in primary hfRPE induced high expression levels of RPE-specific genes, triggering cell reprogramming to RPE epithelial cells characterized by hexagonal shape with junctional multiplexes (Adijanto et al., 2012). Additionally, it has also been observed that miR-148a induced reduction in *ZO-1* expression and disruptions to RPE morphology (Takayama et al., 2016). Moreover, the expression of miR-20b/106a and miR-222/221 families are likely to be essential for maintaining RPE barrier properties and function and have crucial functional roles in RPE homeostasis (Ohana et al., 2015). As part of the BRB, the RPE transports water, ions, and metabolic waste from the subretinal space to the choriocapillaris (Marmor, 1990; Hamann, 2002), where it takes up nutrients such as glucose, vitamin A, retinol, and fatty acids to the photoreceptors (Ban and Rizzolo, 2000; Marmorstein, 2001). To regulate transport across the RPE, various pumps, channels, and transporters are distributed specifically to either the apical or the basolateral membrane. In particular, it has been shown that Kir7.1 is the most abundant potassium channel localized at the apical membrane in the RPE (Yang et al., 2008). Kir7.1 mediates the interactions between retinal photoreceptors and RPE following transitions between light and dark. Wang et al. (2010) also provided the earliest evidence that miR-204 indirectly suppresses Kir7.1 via *TGF-*β*R2* repression in human RPE and thus likely limits potassium efflux across the RPE apical membrane. Additionally, the Kir7.1 channel is functionally coupled to other apical membrane potassium transporters, and the recycling capacity of this channel is maintained by high levels of miR-204 expression. In this context, supporting these observations, miR-204^–/–^ mice showed a reduced efflux of K^+^ transport, which accumulates in the RPE and induces cell swelling and alteration of subretinal space (Zhang et al., 2019). Furthermore, in several studies, it has been shown that miR-204 controls the expression of its host gene, namely, *TRPM3*, which conducts Ca^2+^ and Zn^2+^ ions in renal cell carcinoma (Hall et al., 2014). Depletion of miR-204 may also increase *TRPM3* expression, resulting in general alterations to ion transportation in the RPE. On the other hand, TRPM3 also cooperates with ZO-1 to maintain junctional permeability and barrier function at the apical membrane and to sense light-induced Ca^2+^ levels in the photoreceptor matrix during the visual cycle (Zhao et al., 2015). The latter suggests that the loss of miR-204 could alter the homeostasis of RPE through multiple mechanisms. Other miRNAs have also been identified as crucial regulators of ion channels. Noteworthy among them, Naso et al. (2020) demonstrated that miR-211-mediated inhibition of *Ezrin* releases a Ca^2+^ microdomain flux into cells through potentiation of TRPML1 channel sensitivity, a lysosomal cation channel implicated in lysosomal biogenesis and function (Gomez et al., 2018). miR-211^–/–^ mice show severely compromised vision characterized by an accumulation of phagolysosomes which contain poorly processed POS in the RPE and cone dystrophy (Naso et al., 2020). Confirming the central relevance of miRNAs in RPE homeostasis, miRNAs also take part in cellular responses to environmental stresses such as hypoxia, oxidative stress, and inflammation. Remarkably, oxidative stress stimulates the production of miRNAs that play a role in connecting the antioxidant defense system with imbalances in redox state. Among those, miR-626 represses *Keap1*, which results in the promotion and activation of Nrf2-dependent antioxidant signaling to protect RPE against oxidative stress (Xu et al., 2019). In line with this finding, Lin et al. (2011) showed antiapoptotic effects of miR-23 against oxidative injury through regulation of *Fas*, suggesting the relevance of miR-23 as a key regulator of ROS-mediated cell death/survival. Finally, miR-30b has been reported to increase the cellular antioxidant defense against oxidative stress by targeting the catalase gene. Conversely, miR-144 has recently been described to exert its apoptotic function by targeting *Nrf2*. In response to oxidative stimuli, miR-144 directly represses *Nrf2*, causing reductions to endogenous levels of glutathione and leaving RPE cells susceptible to oxidative stress (Jadeja et al., 2020). These changes in miRNA expression levels suggest dual roles for miRNAs, giving rise to protective or pathological apoptotic phenotypes. Remarkably, Nrf2 also protects retinal tissues from vascular inflammation. A number of additional studies hint at miRNAs playing a role in counteracting the chronic neovascularization in RPE by regulating the expression patterns of proangiogenic factors. For example, miR-9 has been demonstrated to target *Srpk1* gene, controlling the alternative splicing of angiogenic *VEGF165* in human RPE cells under oxidative stress (Yoon et al., 2014). Notably, overexpression of miR-9 effectively reduces the mRNA levels of proangiogenic *VEGF165a* in hypoxia, while the inhibition of miR-9 decreases antiangiogenic *VEGF165b* mRNA levels (Figure 2). These findings further imply that alterations in specific miRNAs (listed in the Table 2) may be used as pathophysiological biomarkers and, additionally, that the precise control of their expression patterns could offer therapeutic strategies to counteract the onset and progression of diverse retinal inherited disorders.

**TABLE 2:** miRNAs involved in the regulation of RPE homeostasis.

miRNA	Target	Function	References
miR-204	TGFβR2, SNAIL2	Regulation of claudins and Kir 7.1 in RPE physiology	Wang et al. (2010)
miR-211	EZRIN	Potentiation of TRPML1 channel sensitivity	Naso et al. (2020)
miR-128/miR-148	ABCA1	Reduction of cellular cholesterol efflux	Hou et al. (2013)
miR-21	p21, Cdc25A, DAXX PPARα	Influence on p53 pathway in retinal microglia Inflammation amelioration	Chen et al. (2017); Morris et al. (2020)
miR-382	NR3C1	Acceleration of intracellular ROS generation	Chen et al. (2020a)
miR-144	NRF2	Worsening of oxidative stress	Jadeja et al. (2020)
miR-626	KEAP1	Protection of RPE cells from oxidative injury	Xu et al. (2019)
miR-195	BCL-2	Oxidative stress increase	Liu et al. (2019)
miR-302a/miR-122	Unknown	Induction of neovascularization after oxidative stress	Oltra et al. (2019)
miR-34a	SIRT1 HSP70	Reduction of oxidative stress tolerance Cryoprotective effect by refolding proteins misfolded in the presence of oxidative stress	Ryhanen et al. (2009); Tong et al. (2019)
miR-601	CUL3	Protection of RPE cells from hydrogen peroxide	Chen et al. (2019)
miR-103	NKILA	Protection of RPE cells from hypoxia	Zhou et al. (2018)
miR-125b	HK2, 15-LOX, SYN-2	Prevention of oxidative stress	Lukiw et al. (2012)
miR-1307/miR-3064/miR-4709/miR-3615/miR-637	KLHl7, RDH11, CERK1, AIPL1, USH1G	Regulation of RPE oxidative stress	Donato et al. (2018)
miR-374a	FAS	Protection of RPE cells against oxidative conditions	Donato et al. (2018)
miR-17	MnSOD, TRXR_2_	Aggravation of oxidative RPE damage	Tian et al. (2016)
miR-23a	GLS1	RPE cells sensitization to H_2_O_2_	Li D.D. et al., 2016
miR-30	Catalase	Antioxidant defense reduction	Haque et al. (2012)
miR-23	FAS	Protection of RPE cells against oxidative damage	Lin et al. (2011)
miR-20/miR-126/miR-150/miR-155	VEGF-A, PDGFβ, NF-κβ, Endothelin, p53	Protection of RPE cells against oxidative stress	Howell et al. (2013)
miR-340	iASPP	Affection of UVB-mediated RPE cell damage	Yan et al. (2018)
miR-141	KEAP1	Protection of RPE from UV radiation	Cheng et al. (2017)
miR-217	SIRT1/HIF1A	Inflammation amelioration	Xiao and Liu (2019)
miR-15a	ASM, VEGF-A	Regulation of proinflammatory pathways	Wang et al. (2016c)
miR-27	TLR4	Inflammation strengthening	Tang et al. (2018)
miR-22	NLRP3	Protection of RPE inflammatory damage	Hu et al. (2019)
miR-155	MC5R, BACH1, SHIP1, CEBPB, IKK	Modulation of inflammatory response	Kutty et al. (2010a), Muhammad et al. (2019)
miR-146a	IL-6, IL-8, TRAF6, IRAK1	Expression regulation of inflammatory genes	Hao et al. (2016)
miR-223	NLRP3	Negative inflammation regulation	Sun et al. (2020)
miR-138	SIRT1	Negative effect on vascular function	Qian et al. (2019)
miR-9	SRPK-1, CEBPA, CEBPB	Biomarker for chronic neovascularization Importance in maintaining RPE cell function	Kutty et al. (2010b, 2013), Yoon et al. (2014)
miR-93	VEGF-1	Choroidal neovascularization involvement	Wang et al. (2016b)
miR-31/miR-150	VEGF, HIF, PDGFβ	Dynamic regulation of neovascularization	Shen et al. (2008)
miR-200	ZEB1, ZEB2, FLT1	CNV involvement	Chung et al. (2016)

## Visual Cycle in the RPE

Vision relies on the functional interaction between RPE and photoreceptors. RPE takes an active part in the visual cycle, as it expresses the enzymes required for reisomerization of the 11-*cis* chromophore from all-*trans*-retinal, which is essential for initiating the photoreceptor response to light. All-*trans*-retinal is reduced to all-*trans*-retinol within the photoreceptors and then transported to the RPE, where it is esterified by LRAT (Saari and Bredberg, 1989; Ruiz et al., 1999). The all-*trans*-retinyl ester product is then isomerized by RPE65 and hydrolyzed to release 11-*cis-*retinol (Redmond et al., 2005). Importantly, phagocytosis and autophagy are crucial cellular mechanisms required for maintaining RPE/PRs homeostasis and supporting the visual system. Light exposure to photoreceptors is accompanied by photo-oxidation of proteins and phospholipids of the outer segments (Beatty et al., 2000). To maintain their function, the POS are shed on a circadian basis (LaVail, 1976, 1980) and phagocyted by the RPE, where they fuse with lysosomes for the degradation and recycling of the ingested POS cargo (Anderson et al., 1978). Alterations to any of the above activities of the RPE is thought to be the primary cause in retinal pathologies, including AMD, because the RPE is vital for the health, survival, and function of the adjacent retinal PRs and choroid (Nandrot et al., 2004, 2006). Remarkably, oscillations in genes related to these cellular processes were observed to respond rapidly to changes in the light environment supporting combinatorial light-dependent and circadian-mediated regulation of visual POS turnover. Phagocytosis and degradation of POS occurs by a non-canonical form of autophagy termed LC3-associated phagocytosis (LAP), which exploits the effectors of autophagy in the process of phagocytosis (Kim et al., 2013; Ferguson and Green, 2014). Importantly, during LAP process, the phagocytic machinery takes advantage of autophagy components Atg5, Atg7, Atg3, Atg12, and Atg16l1 for the lipidation of LC3, resulting in LC3 recruitment to the phagosome and uses a BECN1-PIK3C3/VPS34 complex lacking Atg14. LAP is also independent of the autophagy preinitiation complex consisting of ULK1/Atg13/FIP200 (Kim et al., 2013). Engulfed POS enters the phagosome on a daily basis, the Atg12-Atg5-Atg16L1 complex is recruited, as is the lipidated form of LC3 (LC3-II). Only then does the lysosome fuse with the phagosome forming the phagolysosome leading to degradation of the POS cargo (Ferguson and Green, 2014).

### The MicroRNAs as Key Players in the Phagocytosis and Cell Clearance

**FIGURE 3:**
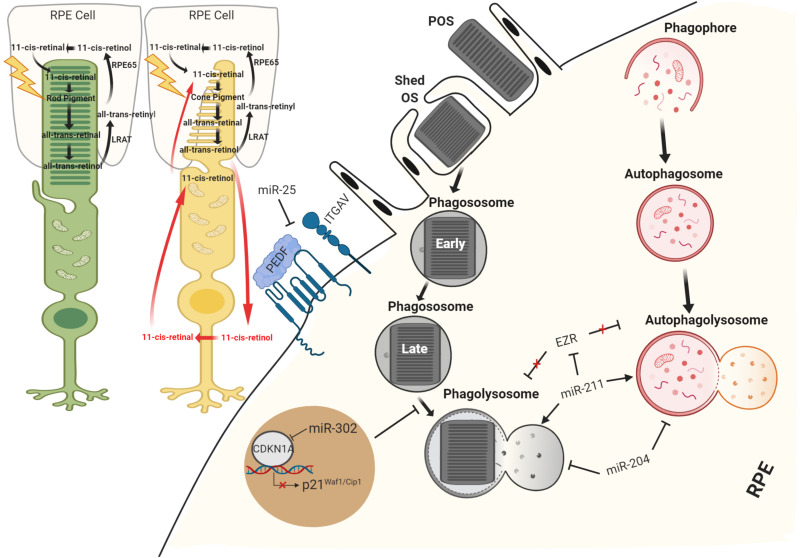
Schematic view of miRNA regulation in RPE recycle. miRNAs have been demonstrated to function both as supporters of visual system and as important regulators of RPE homeostasis maintaining processes, phagocytosis, and autophagy.

The relevance of miRNAs in RPE phagocytosis and cell clearance soon became evident from studies on the RPE from Dgcr8 cKO mice. Specifically, these mice displayed significant decreases in the phagocytotic process accompanied by the presence of lipid droplet-like structures and a lower recovery rate of the visual chromophore 11-*cis*-retinal in the RPE (Sundermeier et al., 2017). Importantly, the daily phagocytosis of POS requires flexibility and the correct orientation of the cytoskeleton in the RPE (Law et al., 2015); uptake and intracellular processing of extracellular material are mediated by both actin and myosin, as well as microtubules for the fusion of lysosomes and phagosomes (Stossel, 1977). Several studies identified Ezrin and ezrin–radixin–moesin (ERM)-binding phosphoprotein 50 (EBP50) as the complex responsible for the polarization dynamics of the RPE microvilli (Bonilha et al., 1999; Kivela et al., 2000; Nawrot et al., 2004). Notably, at the apical surface of RPE, Ezrin and EBP50 can associate with the cellular retinaldehyde-binding protein (CRALBP) in a complex necessary for the release of 11*-cis*-retinal or uptake of all-*trans*-retinol (Bonilha et al., 2004). Preliminary evidence for the role of miRNA in phagocytosis was reported by Murad et al. (2014) showing that inhibition of miR-184 results in the upregulation of *Ezrin* gene and causes downregulation of the phagocytosis in ARPE-19 cells, altering RPE homeostasis. Interestingly, RPE primary culture from AMD donors also showed downregulation of miR-184, consistent with an altered visual cycle (Murad et al., 2014). Moreover, recently, an interesting study showed that miR-302d-3p interrupts phagocytosis in the RPE cells. The latter was correlated to a role for miR-302d-3p in promoting RPE dedifferentiation by targeting *P21^Waf1/Cip1^*, a cyclin-dependent kinase inhibitor (Jiang et al., 2018). A number of additional miRNAs have been implicated to phagocytosis process, including miR-410 (Choi et al., 2017), miR-194 (Cui et al., 2019), and miR-25 (Zhang et al., 2017a) (Figure 3). miR-25 was the first miRNA to be well characterized *in vivo*. Oxidative stress promotes miR-25 expression in the RPE cells by STAT3 signaling, this in turn decreases phagocytosis through direct miR-25-mediated targeting of *IGTAV* and *PEDF*. Interestingly, subretinal injection of antago-miR-25 in a rat model of retinal degeneration, induced by sodium iodate (SI)-mediated oxidative stress, regressed the impairments of phagocytosis and also ameliorated the RPE degeneration (Zhang et al., 2017a). Similarly, *in vitro* studies suggest that inhibition of miR-410 is also able to induce phagocytic capabilities by increasing gene and protein expression of RPE-specific factors (Choi et al., 2017). A recent study has also reported that miR-204 is also involved in phagocytosis and in the processing of phagocyted POS by lysosomes. More specifically, miR-204^–/–^ mice are characterized by retinal degeneration caused by the incomplete degradation of POS and lysosomal accumulation of rhodopsin (Zhang et al., 2019). The study revealed that the absence of miR-204 causes an increase in *Rab22a*, an inhibitor of endosomal maturation. Rab22a is essential to the maturation of early autophagosomal/endosomal intermediates that do not fuse with lysosomes. Thus, miR-204 reduction determines an accumulation of undigested POS, showing a blockage of phagolysosome activity (Zhang et al., 2019). Mice lacking miR-204 also showed inefficient transportation of 11-*cis*-retinal from RPE to the photoreceptors associated with significant reduction to specific gene expression levels (i.e., *RPE65*, *LRAT*, and *TTR*) required for visual pigment regeneration (Zhang et al., 2019). Importantly, severe retinal defects were observed in patients affected by a dominant mutation in miR-204, as the genetic cause of a retinal degeneration associated to other ocular manifestations (Conte et al., 2015). A number of additional miRNAs have been validated to modulate autophagy and recycle of visual components in the RPE (Table 3). miR-30 was also found to play an important role in autophagy by downregulating *Beclin 1* and *Atg5* expression (Yu et al., 2012). Moreover, Lian et al. (2019) demonstrated that miR-24 plays an important role in regulating autophagy in the RPE. This study identified *chitinase-3-like protein 1* (CHI3L1) as a main target. Importantly, *CHI3L1* was suggested to activate the AKT/mTOR and ERK pathways resulting in aberrant autophagy and RPE dysfunction (Lian et al., 2019). Notably, the circadian clock and light are the major regulators of POS phagocytosis and autophagy in the RPE. Several miRNAs were identified to play a role in modulating circadian timing and light-induced responses (Xu et al., 2007; Huang et al., 2008; Pegoraro and Tauber, 2008). Among these, miR-211 is of particular importance because it shows a light-dependent expression pattern (Krol et al., 2010) and it is relevant in homeostasis maintenance of retinal cells through diurnal lysosomal biogenesis in the RPE (Naso et al., 2020). miR-211^–/–^ mice are characterized by defective lysosomal biogenesis and degradative capacities, resulting in the accumulation of POS within the RPE. While miR-211 activation induced autophagy and rescued the anomalies in lysosomal biogenesis. This study also showed that miR-211 targets and represses *Ezrin*; this in turn promotes a Ca^2+^-mediated activation of calcineurin, which leads to TFEB nuclear translocation, thus inducing the expression of lysosomal and autophagic genes (Naso et al., 2020).

**TABLE 3:** miRNAs in recycling visual pigments.

miRNA	Target	Function	References
miR-137/miR-363/miR-92/miR-25/miR-32	RPE65	Negative regulation of visual cycle	Masuda et al. (2014), Zhang et al. (2017a)
miR-124	SOX9, LHX2	Visual cycle inhibition	Masuda et al. (2014)
miR-183/miR-96/miR-182	BDNF	Protection against retinal light injury	Li et al. (2015)
miR-204	RAB22A	RPE endolysosome function alteration	Zhang et al. (2019)
miR-211	EZRIN	Modulation of lysosomal biogenesis and retinal cell clearance	Naso et al. (2020)
miR-184	EZRIN	Efficiency of POS uptake increase	Murad et al. (2014)
miR-410	RPE65	Induction of phagocytic capabilities	Choi et al. (2017)
miR-302	CDKN1A	RPE phagocytosis inhibition	Jiang et al. (2018)
miR-25	IGTAV, PEDF	Phagocytosis decrease	Zhang et al. (2017a)
miR-29	LAMTOR/p18	Autophagy enhancement	Cai et al. (2019)
miR-1273	MMP2, MMP9, TNF-α	Autophagy/lysosome pathway modulation	Ye et al. (2017)
miR-617	MYO7A	Lysosome movement in RPE cells influence	Tang et al. (2015)

## miRNA Functions in RPE Diseases

**FIGURE 4:**
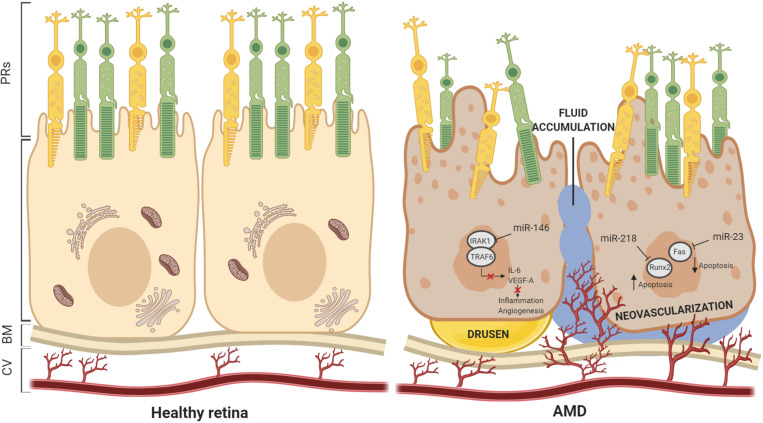
Schematic view of miRNAs dysfunction in AMD. miRNAs have been demonstrated to play key roles in maintaining the structure and functionality of Bruch’s membrane and RPE cells. Incidence of drusen and neovascularization, fluid accumulation, and vascular leakage are often consequences of miRNAs dysfunction. In this scheme, we report examples of miRNAs dysregulation associated with AMD pathogenesis. BM, Bruch’s membrane; CV, choroidal vasculature.

Age-related macular degeneration is a multifactorial, degenerative disease and the most common cause of vision impairment and blindness in the elderly population, with approximately 30–50 million people affected worldwide. The susceptibility to develop “dry” and “wet” AMD forms is dependent on a combination of genetic components and environmental factors. AMD, as well as other forms of retinal degeneration, is often similar and associated with impaired function of the RPE. AMD is characterized by deposits of lipofuscin and extracellular protein aggregates called “drusen” determining progressive dysfunction of autophagy and both RPE and photoreceptor cell death. Interestingly, studies carried out on AMD highlighted that Dicer1 mRNA was reduced in the RPE, but not into neural retina, by 65 ± 3% compared to control eyes, while there was no differences in *Drosha* and *Dgcr8* mRNAs in AMD eyes (Kaneko et al., 2011). To define the relevance of Dicer1 reduction in the RPE, Kaneko et al. (2011) crossed *Dicer1*f/f mice with *BEST1* Cre mice, which express Cre recombinase under the control of the RPE-cell-specific BEST1 promoter. Results showed that these mice displayed RPE cell degeneration in comparison to controls. However, Dicer1 dysregulation in these mice was associated to Dicer1 capacity of silencing toxic *Alu* transcripts rather than miRNA biogenesis (Kaneko et al., 2011). To verify the hypothesis that miRNA dysregulation is implicated in the pathophysiology of AMD, global expression profiles of miRNAs have been investigated. Several studies examined + circulating miRNAs as well as tissue-specific miRNA expression patterns from age-matched control and affected individuals. The results show a number of discrepancies in the number and type of miRNA identified, probably due to the procedures applied to quantify miRNAs and selection of analyzed samples. In spite of these discrepancies, it is evident that most of miRNAs participated to oxidative stress, inflammation, lipid metabolism, and angiogenesis. Remarkably, miRNA expression profiles in both AMD mouse and rat models exhibited overlapping results, suggesting that the main role of miRNAs in RPE is to protect against oscillations in gene expression and counteract environmental stress, in an effort to preserve RPE cellular homeostasis (Berber et al., 2017). Table 4 lists the most commonly reported miRNAs identified in pathological RPE conditions. Interestingly, the most frequently altered expression identified was that of miR-146a, which occurred in both “dry” and “wet” AMD patients and was found in the RPE of different AMD animal models. Remarkably, *in vivo* and *in vitro* studies of specific miR-146a functions demonstrated key roles including repressing expression of *IL-6* and *VEGF-A* genes and inactivating the NF-κB signaling pathway in the RPE. The latter, together with the gain-of function studies for miR-146a, suggests a crucial role for this miRNA in controlling genetic pathways essential to innate immune responses, inflammation, and the microglial activation state, which are major features in the pathogenesis of AMD (Hao et al., 2016). This further suggests that miR-146a may represent a useful disease biomarker and, additionally, a valuable therapeutic target for AMD treatment. Moreover, recent findings reported the role of the epithelial–mesenchymal transition (EMT) in the RPE as a pathological feature of the early stages of AMD (Shu et al., 2020). Remarkably, transforming growth factor-beta (TGFβ) has been identified as a key cytokine orchestrating EMT, and its alteration has been largely associated with onset and progression of several ophthalmological diseases, including AMD (Wang et al., 2019). Interestingly, dynamic changes in expression levels of several miRNAs were associated with TGF-β signaling during EMT in the RPE (Li D.D. et al., 2016). Among these, miR-29b was the most significantly downregulated. The function of the miR-29b was examined in ARPE-19. Additional consequences of miR-29b downregulation included the downregulation of E-cadherin and ZO-1, upregulation of α-smooth muscle actin (α-SMA), and increased cell migration. *In vitro* analyses showed that overexpression of miR-29b was sufficient to reverse TGF-β-mediated EMT through targeting Akt2. In agreement with this, silencing of the Akt2 abolished miR-29b-mediated repression of EMT process (Li M. et al., 2016). Similarly, a detailed study by Jun and Joo (2016) demonstrated that miR-124 was also involved in EMT in the ARPE-19. miR-124 overexpression induced occludin (OCLN) and ZO-1 and repressed α-SMA by directly targeting Ras homology growth-related (RHOG) (Jun and Joo, 2016). Besides miR-29b and miR-124, other miRNAs have been shown to negatively control the EMT. The levels of the ZEB1 protein, a key transcription factor in EMT, in ARPE-19 cells were decreased in the presence of miR-194 duplexes and elevated on miR-194 inhibition. In agreement with these observations, the levels of expression of ZEB1 target genes were reduced in response to miR-194 overexpression as a consequence of alterations in ZEB1 repression. The miR-194 targeting of ZEB1 has also been confirmed *in vivo*. Exogenous administration of miR-194 ameliorated the pathogenesis of proliferative vitreoretinopathy in a rat model (Cui et al., 2019). Accordingly, a recent study also showed that both miR-302d and miR-93 exert their functions in regulating TGFB signaling in the RPE (Fuchs et al., 2020). Altogether, these data highlight a potential use for miRNA: as a promising therapeutic resource for the treatment of ocular disorders. An additional key player involved in AMD and pathophysiology of RPE is miR-211. Indeed, two independent studies based on genome-wide searches for novel AMD risk factors identified miR-211 and its host gene, namely *TRPM1*, as a locus-containing risk factors for AMD (Persad et al., 2017; Orozco et al., 2020). Remarkably, the RPE phenotype in the miR-211^–/–^ mice was similar to that observed in animal models for AMD conditions. Moreover, miR-211 gene delivery via gene therapy was also explored to promote RPE and PR survival in AMD (Cunnusamy et al., 2012). These studies, together with *in vitro* studies demonstrating the function of miR-211 in the physiology of RPE, support that miR-211 is central to RPE homeostasis and, consequently, may serve as a precious molecular tool to counteract AMD disease. A number of additional examples of miRNAs involved in inflammation, oxidative stress, and angiogenesis have already been shown to be potential therapeutic targets for AMD. miR-23a is downregulated in RPE cells from AMD patients, and overexpression of this miRNA in ARPE-19 reduces cell apoptosis by regulating *Fas* (Lin et al., 2011). On the contrary, upregulation of miR-218 accelerates the apoptosis of ARPE-19 cells, negatively regulating *Runx2*. These finding reveal that miR-218/*Runx2* axis could be a therapeutic target for retinal diseases (Yao et al., 2019) (Figure 4).

**TABLE 4:** miRNAs in AMD.

miRNA	Target	Function	References
miR-184	LAMP1, EZRIN AKT2/mTOR	Affection of RPE phagocytosis Prevention of RPE dysfunction and AMD	Murad et al. (2014); Jiang et al. (2016)
miR-34a	TREM2	Drusen formation in AMD	Smit-McBride et al. (2014); Bhattacharjee et al. (2016)
miR-24	CHI3L1	RPE protection from degeneration	Lian et al. (2019)
miR-34	SIRT1, TREM2	Modulating age-related conditions, including AMD	Bhattacharjee et al. (2016); Tong et al. (2019)
miR-30	ITGB3, CRP, PON2, RB1, RPGR, EDNR	Modulating AMD pathogenesis	Haque et al. (2012)
miR-302	CDKN1A	Contribution to the pathogenesis of both atrophic and exudative AMD	Jiang et al. (2018)
miR-155	CFH	Activation of immune-related signaling	Lukiw et al. (2012)
miR-200	ZEB1, ZEB2, VEGFR1	Modulation of pathological ocular angiogenesis	Chung et al. (2016)
miR-20/miR-126/miR-150/miR-155	VEGF-A, PDGFβ, NF-B, endothelin, p53	Protection of RPE cells against oxidative stress	Howell et al. (2013)
miR-9	CEBPA, CEBPB	Maintenance of RPE function	Kutty et al. (2010b)
miR-204	RAB22A	Modulation of endolysosomal and/or autophagy pathways	Zhang et al. (2019)
miR-29	LAMTOR/p18	Contribution to AMD progression	Cai et al. (2019)
miR-223	CCL3, NLRP3, STAT3	Regulation of inflammation during retina degeneration	Fernando et al. (2020)
miR-302/miR-122	VEGF	Regulation of vasculogenesis	Oltra et al. (2019)

## Conclusion

We are in the process of increasing our knowledge on the role of miRNAs as key regulators of RPE homeostasis, function, and survival. miRNAs play an important role in contributing to the precise control of RPE homeostasis in both physiological and pathological states. These insights are opening up avenues to a wide range of novel research areas, including gene-independent medicine, and offers an exclusive class of biomarkers for diagnostic analysis. Recent preclinical studies indicate that miRNAs-based gene therapy is nearing the transition to clinical trial stages. As a single miRNA has the potential to modulate and orchestrate entire molecular programs, future studies are required to shed new light on how they may be safely used as key factors stabilizing and/or resetting the RPE state in pathological conditions. However, recent findings suggest that they are highly promising molecular tools to treat and counteract retinal degeneration including the AMD onset and progression.

## Author Contributions

DI, GG, and IC have contributed to wrote the manuscript and prepared the figures. All authors contributed to the article and approved the submitted version.

## Conflict of Interest

The authors declare that the research was conducted in the absence of any commercial or financial relationships that could be construed as a potential conflict of interest.

## Abbreviations

TNRC6: trinucleotide repeat-containing gene 6A
OTX2: orthodenticle homeobox 2
MITF: melanocyte inducing transcription factor
SOX9: SRY-box 9
TARBP2: TAR RNA-binding protein 2
CTNNBIP1: catenin beta interacting protein 1
Wnt: wingless-type MMTV integration site family member
AKT: protein kinase B
mTOR: mammalian target of rapamycin
PAX6: paired box protein
TGF-βR2: transforming growth factor beta receptor II
CDKN1A: cyclin-dependent kinase inhibitor 1A
RPE65: retinal pigment epithelium-specific 65 kDa
HGF/SF: hepatocyte growth factor/scatter factor
TTR: transthyretin
ZO-1: zonula occludens-1
KIR7.1: inward-rectifier potassium channels
TRPM3: transient receptor potential cation channel subfamily M member 3
TRPML1: mammalian mucolipin transient receptor potential
KEAP1: Kelch-like ECH-associated protein 1
NRF2: nuclear factor erythroid 2-related factor 2
SRPK1: serine–arginine protein kinase 1
VEGF165: vascular endothelial growth factor 165
LRAT: lecithin retinol acyltransferase
ATG: autophagy-related
ATG16l1: autophagy-related 16-like 1
BECN1: Beclin 1
PIK3C3/VPS34: phosphatidylinositol 3-kinase, catalytic subunit type 3
ULK1: Unc-51-like autophagy activating kinase 1
FIP200: FAK family kinase-interacting protein of 200 kDa
STAT3: signal transducer and activator of transcription 3
IGTAV: integrin α V
PEDF: pigment epithelium-derived factor
RAB22A: Ras-related protein Rab-22A
CHI3L1: chitinase-3-like protein 1
ERK: extracellular signal-regulated kinase
TFEB: transcription factor EB
IL-6: interleukin 6
VEGF-A: vascular endothelial growth factor A
NF-κβ: nuclear factor kappa-light-chain-enhancer of activated B cells
TRPM1: transient receptor potential cation channel subfamily M member 1
RUNX2: Runt-related transcription factor 2
SIX3: six homeobox 3
LHX2: LIM/homeobox 2
CHX10: Ceh-10 homeodomain-containing homolog
NRL: neural retina leucine zipper
PDC: phosducin
DCT: dopachrome tautomerase
NOX2: NADPH oxidase 2
LGR4: leucine-rich repeat containing G protein-coupled receptor 4
ATF2: activating transcription factor 2
SOCS3: suppressor of cytokine signaling 3
ZEB: zinc finger E-box -binding homeobox
RhoG: Ras homology growth-related
TGFβ: transforming growth factor beta
ABCA1: ATP-binding cassette transporter A1
Cdc25A: cell division cycle 25 A
DAXX: death domain associated protein
PPARα: peroxisome proliferator activated receptor α
NR3C1: nuclear receptor subfamily 3 group C member 1
BCL-2: B-cell lymphoma 2
SIRT1: NAD-dependent deacetylase sirtuin-1
HSP70: heat shock protein 70 kDa
CUL3: Cullin-3
NKILA: NF-kappaB interacting LncRNA
HK2: hexokinase 2
15-LOX: 15-lipoxygenase
SYN-2: synapsin II
KLH17: Kelch-like family member 7
RDH11: retinol dehydrogenase 11
CERK1: chitin elicitor receptor kinase 1
AIPL1: aryl hydrocarbon receptor interacting protein-like 1
USH1G: Usher syndrome type-1G
MnSOD: manganese superoxide dismutase
TRXR_2_: thioredoxin reductase 2
GLS1: glutaminase 1
PDGFβ: platelet-derived growth factor β
iASPP: inhibitor of apoptosis-stimulating protein of p53
HIF1A: hypoxia-inducible factor 1-alpha
ASM: acid sphingomyelinase
TLR4: Toll-like receptor 4
NLRP3: NOD–, LRR–, and pyrin domain-containing protein 3
MC5R: melanocortin 5
BACH1: BTB domain and CNC homolog 1
SHIP1: SH-2 containing inositol 5 ′ polyphosphatase 1
CEBPB: CCAAT enhancer binding protein beta
IKK: inhibitor of upstream I κ B kinase
IL-8: interleukin 8
TRAF6: TNF-receptor-associated factor 6
IRAK1: interleukin 1 receptor-associated kinase 1
SRPK1: SRSF protein kinase 1
CEBP: CCAAT enhancer binding protein
FLT1: Fms-related receptor tyrosine kinase 1
BDNF: brain-derived neurotrophic factor
LAMTOR: late endosomal/lysosomal adaptor and MAPK and mTOR activator 1
MMP2: matrix metalloproteinase-2
MMP9: matrix metalloproteinase-9
TNF-α: tumor necrosis factor-alpha
MYO7A: myosin VIIA
TREM2: triggering receptor expressed on myeloid cells 2
ITGB3: integrin subunit beta 3
CRP: C-reactive protein
PON2: paraoxonase 2
RB1: RB transcriptional corepressor 1
RPGR: retinitis pigmentosa GTPase regulator
EDNR: endothelin 3
CFH: complement factor H
VEGFR1: vascular endothelial growth factor receptor 1
CCL3: C–C motif chemokine ligand 3.
